# A Timm-Nissl multiplane microscopic atlas of rat brain zincergic terminal fields and metal-containing glia

**DOI:** 10.1038/s41597-023-02012-6

**Published:** 2023-03-21

**Authors:** Camilla H. Blixhavn, Finn-Mogens Š. Haug, Heidi Kleven, Maja A. Puchades, Jan G. Bjaalie, Trygve B. Leergaard

**Affiliations:** Department of Molecular Medicine, Institute of Basic Medical Sciences, University of Oslo, Oslo, Norway

**Keywords:** Brain, Neuroscience

## Abstract

The ability of Timm’s sulphide silver method to stain zincergic terminal fields has made it a useful neuromorphological marker. Beyond its roles in zinc-signalling and neuromodulation, zinc is involved in the pathophysiology of ischemic stroke, epilepsy, degenerative diseases and neuropsychiatric conditions. In addition to visualising zincergic terminal fields, the method also labels transition metals in neuronal perikarya and glial cells. To provide a benchmark reference for planning and interpretation of experimental investigations of zinc-related phenomena in rat brains, we have established a comprehensive repository of serial microscopic images from a historical collection of coronally, horizontally and sagittally oriented rat brain sections stained with Timm’s method. Adjacent Nissl-stained sections showing cytoarchitecture, and customised atlas overlays from a three-dimensional rat brain reference atlas registered to each section image are included for spatial reference and guiding identification of anatomical boundaries. The Timm-Nissl atlas, available from EBRAINS, enables experimental researchers to navigate normal rat brain material in three planes and investigate the spatial distribution and density of zincergic terminal fields across the entire brain.

## Background & Summary

The transition metal zinc is involved in several fundamental neurobiological processes ranging from brain growth and cell differentiation^1–3^ to synaptic functions^4–9^ including plasticity^10^. Most of the zinc is bound to proteins where it has structural or catalytic functions. The remaining, loosely bound or “free”, fraction may have signalling functions^11^. Excess concentrations of “free” zinc can cause cell death^12,13^ and contribute to degenerative diseases and aging^14^. The concentrations of free zinc are therefore controlled by zinc transporters^15^, metallothioneins^16^ and possibly other zinc proteins^17^. Other homeostatic mechanisms control the concentration of “free” copper, which is now also recognized as a signalling molecule^18^, and *iron*^19–21^. Zinc has been implicated in pathophysiological processes underlying brain diseases such as epilepsy^22^, ischemic stroke^23–26^, traumatic brain injury^27–29^, Alzheimer’s^30,31^ and Parkinson’s disease^32^, neuropsychiatric conditions such as major depression^33^ and schizophrenia^34^, and neurodevelopmental disorders including autism spectrum disorders^35,36^. Due to their key roles in neurobiological and neuropathological processes, there is considerable interest in understanding the normal homeostasis and distribution of zinc and other transition metals in the central nervous system. For such research efforts, knowledge about the spatial distribution of zinc and other heavy metals in the brain is important. Several methods are now available to indicate the distribution of free transition metals in the brain, especially fluorescence microscopy of live tissues and cells^37–39^. Nevertheless, autometallographic detection of metals using silver amplification remains a powerful and relevant approach to studies of transition metals in the central nervous system, due to its sensitivity, contrast and spatial resolution.

Timm’s sulphide silver method, introduced by Dr. Friedrich Timm in 1958^40^, is a histochemical staining method broadly selective for several transition metals and heavy metals. Based on precipitation with sulphide, and visualisation of the precipitate by physical development, the method is selective for free or loosely bound metals, but not specific for any one metal. In the nervous system the sulphide (and selenium) silver methods label neuropil^41–43^, neuronal somata^41,43,44^ and glia^43,45^ to varying degrees. The staining of *neuropil* in general corresponds to axonal terminal fields^42,43,46–49^ where it is confined to boutons^47,49–57^ of zinc-containing^58,59^, or *zincergic*^7,60,61^, neurons. Zincergic boutons are defined by activity-induced release of zinc from their synaptic vesicles^62–65^. Most zincergic boutons are glutamatergic^66–70^, some are GABAergic, especially in the brain stem and spinal cord^71,72^. Many glutamatergic boutons are Timm-negative^67,68^. The signalling functions of synaptic zinc have been reviewed by several groups^5–9^.

Timm-staining of neuropil is useful as a neuromorphological marker, in subdividing parts of the telencephalon^43,46,48,73–76^, analysing interstrain^77^ and interspecies differences and homologies^78–85^, following ontogenetic development in normal^57,86,87^ and mutant^88^ strains and investigating experimentally induced synaptic plasticity in developing^89–93^ and adult^61,91,92,94^ brains, including experience-dependent plasticity^61,95^.

Recognizing the broad interest and relevance of Timm’s method for investigating zinc-related phenomena in the brain, we here present a multiplane histological atlas of normal rat brain zincergic terminal fields and metal-containing glia, correlated to cytoarchitecture as revealed by Nissl-staining. The Timm-Nissl atlas may act as a benchmark reference and support for planning and interpretation of experimental studies, particularly for studying the extent and anatomical boundaries between brain (sub)regions in three planes, as well as for planning and interpreting experimental studies which depend on precise regional sampling. The image collection comprises microscopic images of historical series of coronally, sagittally or horizontally cut rat brain sections stained alternately with a modified^43^ Timm’s sulphide silver method and a standard Nissl method. Custom reference atlas overlay images are provided to facilitate interpretation of anatomical locations. The high-resolution images are shared via the EBRAINS research infrastructure (https://search.kg.ebrains.eu/), with options for interactive inspection in a web microscopy viewer.

## Methods

### Overview of methodology and historical context

**Fig. 1:**
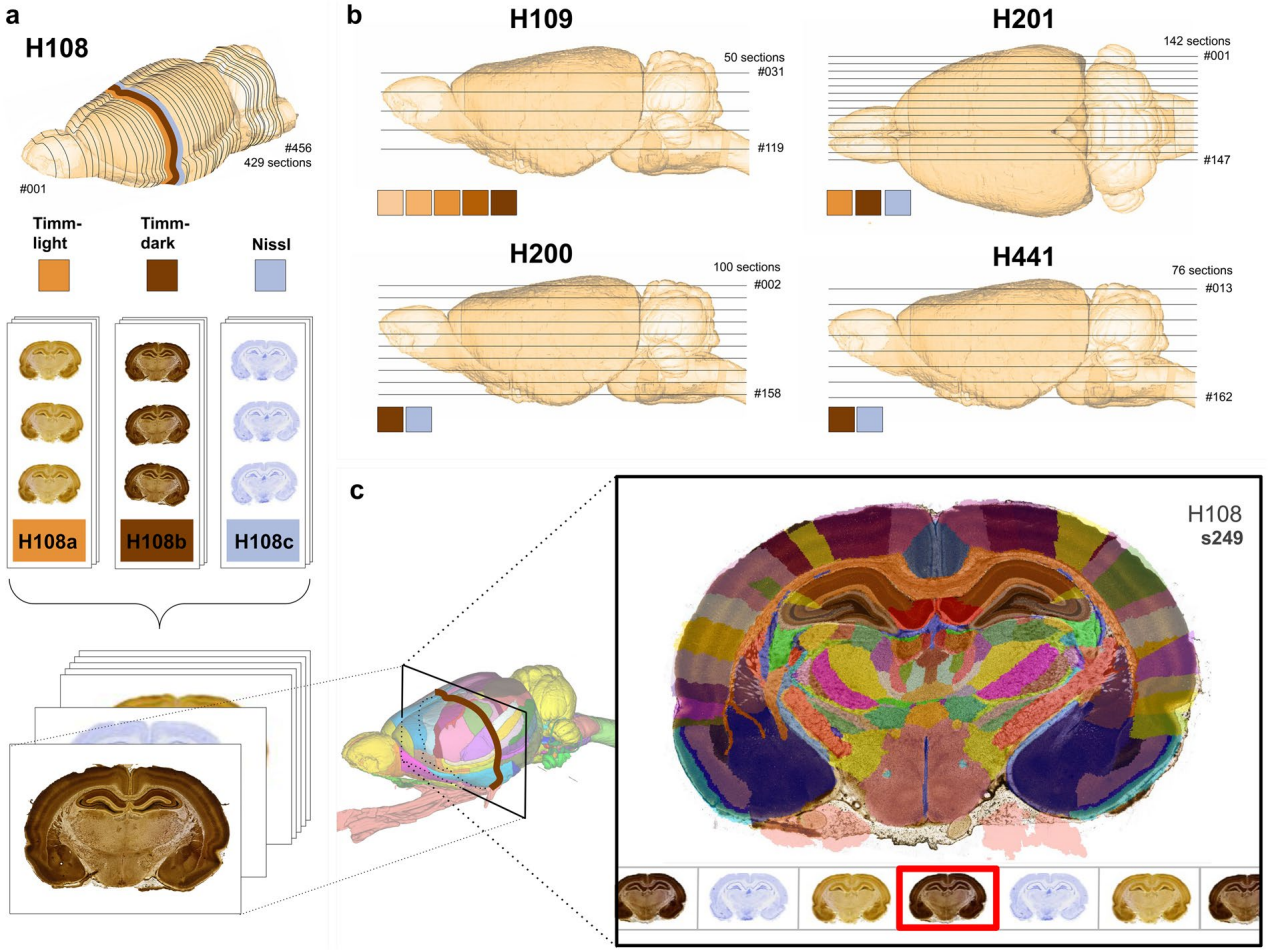
Overview of the rat brain Timm-Nissl atlas. (**a**) Serial coronal rat brain sections, H108, were cryosectioned and stained with the TimmHaug73 modification of Timm’s sulphide silver method (Timm-light or Timm-dark), or Nissl-staining (thionine). (**b**) Sections from the remaining rat brains were stained following the same procedures as exemplified with H108 above in Fig. 1a, although with individual differences according to sectioning plane and staining. H109 (one hemisphere) was horizontally sectioned and split into subseries and stained with Timm-Haug73 modification of Timm’s sulphide silver method in sequences of different time intervals (30–70 minutes). H200 and H441 were horizontally sectioned and split into subseries of Timm-dark and Nissl, while H201 was sagittally sectioned and stained with Timm-light, Timm-dark and Nissl. (**c**) The TIFF images of the different subseries of sections, here exemplified with subject H108 (Timm-light, Timm-dark, Nissl), were pre-processed and spatially registered to the Waxholm Space atlas of Sprague Dawley rat brain v4^100^. Serial section images are shared via the EBRAINS Research Infrastructure, available for interactive inspection in an online viewer tool with atlas overlay.

The Timm-Nissl atlas is based on a historical collection of rat brain sections stained with Haug’s version^43^ of Timm’s method^40^, in the following referred to as Timm-Haug73, developed for bulk-staining of serial sections, and used to comprehensively map the Timm-staining pattern in the rat brain^43^ and specifically the hippocampal formation and parahippocampal region^46,48^, henceforth referred to as the hippocampal region^96,97^. The protocols used to stain the sections included in the present collection are summarised below. For further details the original publication^43^ should be consulted. The Technical Validation chapter below includes a comparative discussion of the staining achieved using the Timm-Haug73 version with the results obtained with other frequently used versions of the method. Figure 1 shows key steps of the workflow from rat brain sectioning to digitised section images with atlas-registered overlays available in web microscopy viewers, and provides overview of the material included in the collection.

### Animal preparations

The histological sections were derived from five adult albino (Wistar) rat brains (Table 1). All animal procedures were performed in accordance with Norwegian and Danish legislation and institutional practice at the Universities of Oslo and Aarhus in the years 1967–1976. The animals were deeply anaesthetised with ether and Nembutal and perfused through the left ventricle with a solution of 11.7 g Na_2_S and 11.9 g NaH_2_PO4.H_2_O per 1000 ml H_2_O, pH = 7.3–7.4 at room temperature using a 2.0 mm Wassermann cannula elevated to a height of 100–110 centimetres. After 1 minute, flow was reduced to approximately 5–10 ml per minute for 20 minutes. The sulphide treatment is not a fixative, so the brains remained soft. The fresh brains were removed from the scull and frozen with CO_2_ gas. The brains were coronally, horizontally or sagittally cryosectioned at 40 µm with a Dittes Duspiva cryostat and mounted on microscope slides by thawing. The brain of subject H108 (coronal series) was coronally bi-sectioned and placed with the cut surface down before freezing, causing slightly different section angles in the anterior and posterior brain block, as well as loss of sections at the level of the mesencephalon. After air-drying for 15 minutes to 2 hours, sections were post-fixed in 90% ethanol for 15 minutes, re-hydrated through a series of descending alcohol concentrations and stained within 5–10 minutes.

**Table 1:** Overview of stained brain section images from five adult rats.

Name of dataset	Animal #	Series #	Staining	Orientation	# Images
***Multiplane microscopic atlas of rat brain zincergic terminal fields and metal-containing glia stained with Timm’s sulphide silver method*** 10.25493/T686-7BX	H108	H108a	Timm-light	coronal	144
		H108b	Timm-dark	coronal	139
		H108c	Nissl (thionine)	coronal	146
	H200	H200a	Nissl (thionine)	horizontal	48
		H200b	Timm-dark	horizontal	52
	H201	H201a	Timm-dark	sagittal	47
		H201b	Nissl (thionine)	sagittal	47
		H201c	Timm-light	sagittal	48
	H441	H441a	Timm-dark	horizontal	38
		H441b	Nissl (haematoxylin)	horizontal	38
***Contrast reference images for the Timm-Haug73 modification of Timm’s sulphide silver method*** 10.25493/4K9X-FJW	H109	H109a	Timm, 30 min	horizontal	10
		H109b	Timm, 40 min	horizontal	10
		H109c	Timm, 50 min	horizontal	10
		H109d	Timm, 60 min	horizontal	10
		H109e	Timm, 70 min	horizontal	10

### Timm-Haug73 staining and thionine staining

Every morphologically intact section was saved, and sections from brains H108, H200, H201 and H441 were mounted as 2–3 parallel series (Table 1, see next paragraph for brain H109), each of which were stained either with the Timm-Haug73 method or with thionine or haematoxylin for cytoarchitecture. This was considered to be more instructive than counterstaining Timm-stained sections for cytoarchitecture. Consecutive serial section numbers were assigned from anterior to posterior, medial to lateral, or dorsal to ventral. For some brains, additional series with other staining are not included in the present collection, although their sequence-numbers are reserved. For Timm-Haug73 staining, sections were batch-incubated in the physical developer (for contents and details, see Table 2) in darkness at 24 °C, using a water bath to maintain constant temperature. The developer was prepared with lumps of raw gum arabic (Riedel de Hahn, Oslo, & Bie and Berntsen, Aarhus), as these were found to yield better results than powdered gum arabic. Cleaning glassware with special agents to remove silver and other metals was not part of the procedure published in Haug 1973 but is now often recommended.

**Table 2:** Constituents of the developer solution used for the Timm-Haug73 version.

Stock Solutions	Volume (mL)	grams	g/100 mL(%)	Mol
Gum arabic	8000	4000	50,00	NA
Citrate buffer	100			
Citric acid.H2O		25,50	25,50	
Tri-Na citrate.2 H2O citrate.2H20citrate.2H2O		23,50	23,50	
Citric + citrate				2,01252
Hydroquinone	100	5,67	5,67	
Silver nitrate	50	8,50	17,00	
**Final developer**				
Gum arabic	60	30,00	30,00	NA
Citrate buffer	10	4,90	4,90	0,201
Hydroquinone	30	1,701	1,701	0,154
Silver nitrate	0,5	0,085	0,085	5 × 10^−3^
**Total**	100,50			

When visual comparison to a standard benchmark time series (see below) showed that the intended strength of staining had been reached, the process was halted by thorough rinsing in tap water for at least 5 minutes to remove the viscous developer solution. Sections were dehydrated in alcohol, cleared briefly in xylene and enclosed under cover slips with dammar resin. Adjacent series of sections were stained using thionine or haematoxylin following standard procedures. The staining and differentiation steps, and particularly the time of differentiation, were adjusted by evaluating the contrast between nuclei and Nissl-substance and neuropil (or myelin in white matter) macroscopically or under low magnification.

The complete collection of sections from the right hemisphere of brain H109 were mounted as 7 parallel series (named a-g), which were stained with the Timm-Haug73 version with 10-minute incrementally increased incubation times (a = 30 minutes; g = 90 minutes) in the developer solution. The contrast reference sections were used to 1) benchmark the staining of the whole brain series of sections and ensure similar staining intensity in the strong Timm-staining (Timm-dark) and light Timm-staining (Timm-light) series, and 2) aid the interpretation of Timm labelling by allowing comparison of features across neighbouring sections stained with increasing intensity.

### Microscopic imaging

Brightfield microscopy images from Timm- and Nissl-stained rat brain section series were acquired using a Zeiss Axioscan Z1 slide scanner (Carl Zeiss MicroImaging, Jena, Germany) through a 20 × objective, yielding images with a 0.22 µm/pixel resolution. The white balance was adjusted using the Zeiss ZEN software (v2.3, RRID: SCR_013672) before images were exported in JPEG-compressed Tagged Information File Format (TIFF). After image acquisition, each image series was inspected to ensure correct orientation and serial order of section images, and if necessary corrected by rotating or mirroring the images, using the open source software Nutil^98^ v0.33, RRID: SCR_017183). Sections with poor tissue quality (defined as less than one half section being intact) were excluded but retained their name in the serial order. In the case of subject H108, section numbers were renumbered from anterior to posterior, and the loss of tissue at the level of the mesencephalon was estimated to correspond to 26 sections between section number 268 and 294.

### Registration with a three-dimensional rat brain reference atlas

To provide a starting point for analysis of anatomical location, all images were spatially registered to the three-dimensional Waxholm Space atlas of the Sprague Dawley rat brain^96,97,99,100^ (WHS rat brain atlas, RRID: SCR_017124, Fig. 1c), using the QuickNII software tool^101^ (RRID: SCR_016854). This tool allows the user to create custom made atlas plates, in any plane of orientation, based on the global match of multiple anatomical landmarks and affine transformation (scaling, panning and rotation). This was followed with further refinements by non-linear transformation, using the VisuAlign software tool (RRID: SCR_017978), and validated independently by another investigator. The registration results, specifying the spatial relationship between the images and the WHS rat brain atlas, were stored in JSON format. In the usage notes and figure text, nomenclature from version 4 of the WHS rat brain atlas is used to ensure seamless communication between text and tools. For finer anatomical details, not delineated in the WHS rat brain atlas v4, nomenclature is referenced and/or defined in figures.

## Data Records

Images and associated metadata are shared as two datasets^102,103^ with DataCite DOIs via the EBRAINS platform (https://ebrains.eu/). The datasets consist of a set of files: JPEG-compressed TIFF section images, and atlas registration JSON records, all described in a data descriptor in PDF format, with relevant metadata registered to associate the datasets appropriately within the EBRAINS Knowledge Graph. Table 1 provides an overview of all rat subjects included.

**Fig. 2:**
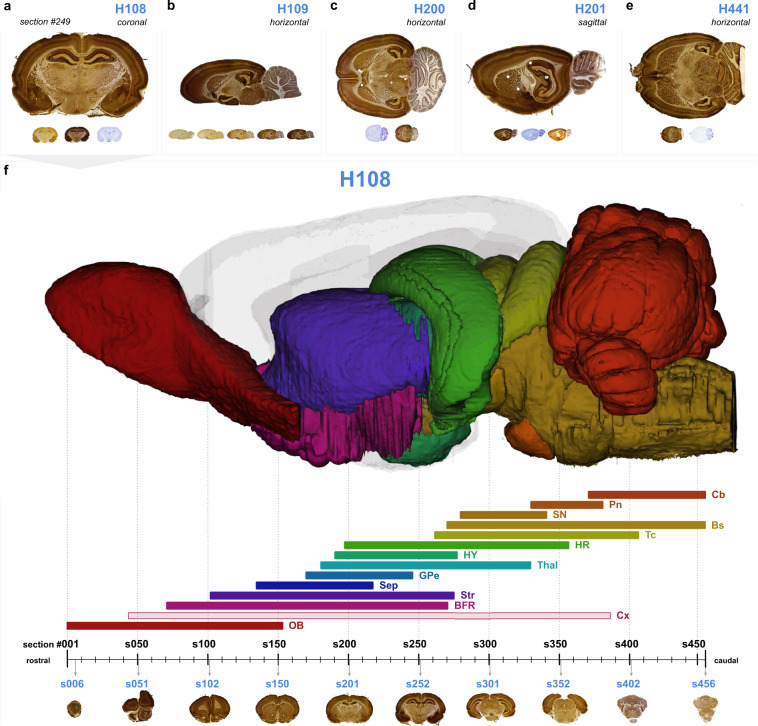
Navigating the rat brain Timm-Nissl atlas. The rat brain Timm-Nissl atlas comprises section images from five rat brains presented in a web-microscopy viewer (via links available from the EBRAINS data card, or via embedded links in the interactive PDF version of this Figure, see Supplementary Figure 1). The content of the (**a**) coronal (H108), horizontal (**b**) (H109), (**c**) (H200), (**e**) (H441) and (**d**) sagittal (H201) series are exemplified with an image of the more intense version of Timm-staining (Timm-dark) as seen in the web viewer. (**f**) Overview of the rostral to caudal distribution of the coronal image series (H108), relative to surface rendered brain regions in the Waxholm Space atlas of the Sprague Dawley rat brain (WHS rat brain atlas, with custom made colour coding)^96^,^100^. Colour coded bars indicate the rostrocaudal range of section numbers distributed along the horizontal axis, that include the different brain regions visualised above. Light blue numbers indicate the section number and rostrocaudal position of the thumbnail section images shown below. The figure can be used as an initial guide to identify the range of coronal sections that contain a region of interest, e.g. the hippocampal region (HR), which is visible in section numbers 200–360. WHS rat brain atlas v4: BFR, basal forebrain; Bs, brain stem; Cb, cerebellum; Cx, cerebral cortex; GPe, globus pallidus, external segment; HR, hippocampal region; HY, hypothalamus; OB, olfactory bulb; Pn, pontine nuclei; Sep, septal region; SN, substantia nigra; Str, striatum; Tc, tectum; Thal, thalamus.

The first data set^102^ “Multiplane microscopic atlas of rat brain zincergic terminal fields and metal-containing glia visualised with Timm’s sulphide silver method” consists of TIFF images of coronal, horizontal and sagittal sections obtained from four adult albino (Wistar) rat brains (H108, H200, H201, H441), alternatingly stained with Timm-Haug73 or with Nissl-staining (Fig. 2). The file names consist of a subject name (H followed by three digits), a letter in serial order (_a, _b, or _c) sample name (_Timm-dark, _Timm-light, or _Nissl), orientation (_coronal, _horizontal, or _sagittal) and serial section number (_s followed by three digits). Each subject image series (H108, H109, H200, H201, and H441) contains between 50 and 429 images and covers all major brain regions. All images were registered to the WHS rat brain atlas yielding one JSON file per subject image series, with a naming convention of subject name followed by orientation. Links to the web-microscopy viewer tool LocaliZoom were created by the EBRAINS Share data service, and are available from the EBRAINS dataset landing pages (see, also ‘usage notes’ below).

**Fig. 3:**
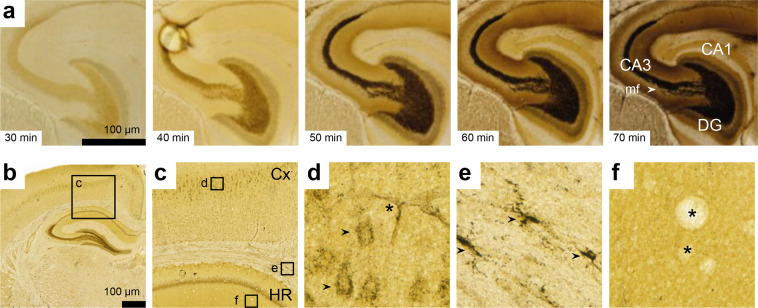
Technical validation of staining intensity and stained features in the Timm-Nissl atlas. (**a**) Timm-Haug73 Contrast Reference Images: Magnified view of the hippocampal region (HR) from 5 rat brain sections (H109, sections #049–053) stained with Timm-Haug731 modification of Timm’s sulphide silver method in sequences of different time intervals (30–70 minutes). (**b**) Overview image with inset c (H108a Timm-light coronal series, section #230). (**c**) Magnified view of cerebral cortex (Cx) and HR with insets indicating positions of different distinct features visualised with Timm-Haug73: (**d**) Neuronal perikarya (black arrows, note vasculature indicated by asterisk); (**e**) Glial cells (black arrows); (**f**) Neuropil (note air bubbles indicated by asterisks).

The second dataset^103^ “Contrast reference images for the Timm-Haug73 modification of Timm’s sulphide silver method” consists of images of horizontal sections from one right rat brain hemisphere (H109) stained using the Timm-Haug73 modification with development times varying from 30 to 70 minutes (a-e, see Fig. 3a). The file names consists of a subject name (H109), a letter in serial order (_a, _b_,…, or _e) sample name (_Timm), indication of development time (30–70) and orientation (horizontal).

## Technical Validation

Timm’s method has been implemented in several versions that yield somewhat different results. To aid the interpretation of the present collection of Timm-Haug73 stained images, we here review some technical topics of importance for interpreting different modifications of Timm’s method. Next, we discuss the accuracy of techniques used to assemble the sections into a brain-wide microscopic Timm- and Nissl-stain atlas, including the anatomical image registration to the WHS rat brain atlas and the custom atlas overlays provided with each brain section.

### The chemical foundation of sulphide silver (and related selenium-) methods

In a generic Timm’s method, metals are precipitated *in situ* by treating biological material with sulphide, whereafter superfluous sulphide is washed out and the material subjected to physical development^40,104^. A physical developer contains silver ions, a reducing agent for converting silver ions to elementary silver and substances to retard the redox-reaction in the solution at large. The metal sulphides, e.g. ZnS, are converted to Ag_2_S, which catalyses further reduction of dissolved Ag^+^ to Ag^0^. The process then continues until it is interrupted or the developer is exhausted, while the staining changes from light yellow towards black (Fig. 3a). Whether the rate of change depends on the concentration of Timm-stainable metals^43,105^ has not been quantitatively investigated. Endogenous or exogenous transition metals reacting with sulphide may be visualised, as long as they are not hidden within proteins or other endogenous ligands and occur in sufficient concentrations. In the central nervous system, Timm’s method reveals the distribution of selected sulphide silver stainable metal species with high spatial resolution and contrast.

### Free vs bound metal fractions

The fractions of zinc which are free, or available, to react with the sulphide in Timm’s method, or with exogenous chelating agents, have been given many names: Free^106–108^, available^109^, histochemically reactive^110^, chelatable^24,111,112^, exchangeable^113^, mobile^114,115^, loosely bound^116,117^ and - for iron - labile iron pool (LIP)^118^, labile cell iron (LCI)^119^ and non-heme iron^120^. Free metal ions are always coordinated by water molecules or other ligands, as illustrated for zinc^121^. The distinction between free and bound metals is methodologically defined, i.e. by the particular chelators and conditions applied in each experiment^106^. Although “free” or “mobile” suggest small-molecular ligands, the fluorophore TSQ was recently found to fluoresce while bound in ternary TSQ-Zinc-protein complexes. As such, the endogenous ligands need not always be low-molecular or mobile, as long as they allow the metal to react with the exogeneous ligand used in the experiment^17,122–124^.

### Different Timm-staining patterns in the central nervous system

In Timm’s method, exogenous sulphide competes for metals with a mixture of endogenous ligands^16,17,122–126^. Different free metal fractions may be selected by changing the concentration of sulphide ions^127,128^, or the duration of sulphide treatment^53^. This may explain differences in staining pattern between the following three versions of Timm’s method. The Timm-Haug73 version stains neuropil, neuronal perikarya and glia after perfusing with a 1.17% solution of sulphide at pH 7.3–7.4. The Timm-Danscher81 (neoTimm) version eliminates staining of neuronal perikarya and most of the glial staining after a 12-times lower sulphide concentration and a very short perfusion-time of 7 minutes. The version from Brun and Brunk^44,127^ enhances staining of neuronal perikarya and glia and downplays that of neuropil, by increasing the concentration of sulphide ions.

For the Timm-Haug73 version^43^, the procedures were tuned to bring out “morphologically plausible structures”, leaving the question of chemical selectivity or specificity for later work. Perfusion with sulphide was chosen to achieve immediate *in situ* precipitation of the metal sulphides. Cryostat-sections were chosen over paraffin sections, as the latter all but abolished Timm-stainability in perikarya and glia (apparent also from illustrations in references^54,129^), an effect which has been attributed to oxidation of sulphide^130^. As aldehyde fixation also reduced the staining in glia and neuronal perikarya, series from non-aldehyde-fixed brains were selected for the original publication and for the present Timm-Nissl atlas. In the two-dimensional images from 40 μm thick cryostat sections, cytological details are obscured where neighbouring stained structures are densely packed and the sections heavily stained. This was partly overcome by routinely preparing a lightly stained (Timm-light) and a more darkly stained (Timm-dark) series, which also accommodates large differences in staining density between different parts of the telencephalon. The effect of different development times is illustrated in detail by subject H109 (Fig. 3a). Timm-stained cell layers (Fig. 3a, 50 min, granule cell layer in the dentate gyrus), single perikarya (Fig. 3d) and glial cells (Fig. 3e) are often best seen in moderately stained sections (Timm-light), while some neuropil layers require longer development times (Timm-dark; Fig. 3a, 60 and 70 minutes). With subjects H108 and H201, one may compensate for this by switching between adjacent Timm-light and Timm-dark sections (Fig. 2a,d).

Some examples of regional and cytological features are given here (Fig. 3a–f). As a supplement to the present atlas, the original publications offer more detailed cytological^43^ and regional^43,46,48^ descriptions. Neuronal perikarya are stained in all parts of the central nervous system (Fig. 3d). Regional distribution or inter-brain variation of this staining were not systematically studied. The staining of glial cells (Fig. 3e) is regionally differentiated; prominent in parts of the brainstem^43^ and sparse in grey matter of the telencephalon. Timm-stained glial cells were described and provisionally classified into six types^43^. Interestingly, numerous Timm-stained glial cells appear in rat cortical areas undergoing anterograde axonal degeneration^49,89,94^.

**Fig. 4:**
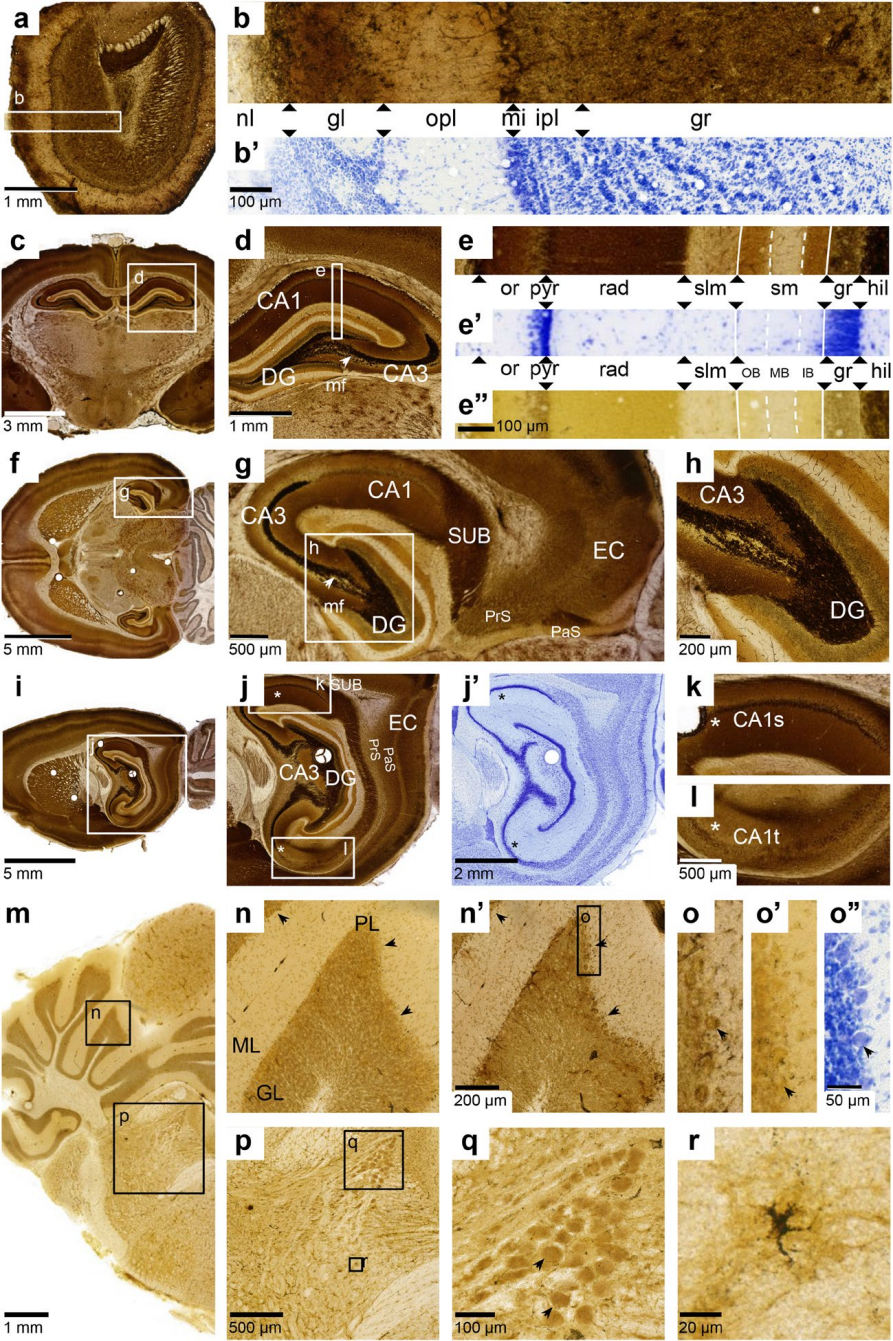
Regional characteristics of the rat brain Timm-Nissl atlas. (**a**) Olfactory bulb (H108b, Timm-dark coronal series, section #042), (**b**) magnified view of Timm-dark (above) and **b’**) Nissl (thionine, below) series visualising different layers of the main olfactory bulb^174^ (MOB): olfactory nerve layer (MOBnl); glomerular layer (MOBgl); outer plexiform layer (MOBopl); mitral layer (MOBmi); inner plexiform layer (MOBipl); granule cell layer (MOBgr). (**c**) Coronal view of hippocampal region^96,97^ (HR) with inset d (H108b, section #237), (**d**) Magnified view, with inset e, of HR including: *cornu Ammonis* 1 and 3 (CA1, CA3), dentate gyrus (DG) and mossy fibres (mf, white arrow), (**e**) Timm-dark (90° rotation, counter-clockwise), magnified view of different layers of CA1^176^: *stratum oriens* (or); *stratum pyramidale* (pyr);* stratum radiatum* (rad); *stratum lacunosum-moleculare* (slm); and DG: outer, middle and inner band of the molecular layer (mol); granular layer (gr); polymorph layer/hilus (hil). e’) Nissl (thionine), and e”) Timm-light. (**f**) Horizontal view of HR with inset g (H200b, Timm-dark sagittal series, section #071), (**g**) Magnified view, with inset f, of HR including CA1–3, DG, mf (white arrow), subiculum (SUB), presubiculum (PrS), parasubiculum (PaS) and entorhinal cortex (EC), (**h**) Magnification of border between CA3 and DG, (**i**) Sagittal view of HR with inset j (H201a Timm-dark sagittal series, section #046), (**j**) Magnified view, with inset k and l (asterisks indicating a septotemporal gradient), of the HR including CA3, DG, SUB, PrS, PaS and EC, (**j**’) Adjacent view of Nissl-stained section and magnified view of (**k**) septal and **l)** temporal part of CA1 (CA1s, CA1t). (**m**) Cerebellum and brainstem^96^ (H108a Timm-light coronal series, section #379) with insets n and p, (**n**) Magnified view of cerebellar cortical layers including molecular layer (ML), granular layer (GL) and Purkinje cell layer (PL, black arrows). (**n**’) Adjacent section (Timm-dark) with inset o, magnification of distinct Purkinje cells (black arrows) in adjacent sections stained with (**o**) Timm-dark, (**o**’) Timm-light, and (**o**”) Nissl (thionine). (**p**) Magnified view of brainstem with inset q and r, (**q**) magnified view of cells (black arrows) in the mesencephalic trigeminal nucleus^43^ and (**r**) a glial cell in the brainstem.

At higher magnification, the staining of neuropil in the telencephalon (Fig. 3f), parts of the brainstem (discernible e.g. in the hypothalamic region in Fig. 4c, see also Figs. 22–23 in reference^43^) and in grey matter of the spinal cord (not shown in the Timm-Nissl atlas, see references^43,131^), is suggestive of boutons. At lower magnification regional differences were qualitatively reproducible despite some quantitative variations as judged by visual inspection. As an example, the outermost stained zone of the molecular layer of the dentate gyrus appeared strongly stained in series from some brains and more weakly stained in series from other brains, when compared to neighbouring areas in the section. The atlas is not designed for densitometric measurements of the Timm-staining.

The Timm-Danscher81 (neoTimm) version^53^ is a modification of Timm-Haug73, using reduced sulphide and hydroquinone concentrations and a stock solution of silver lactate rather than silver nitrate. It replicates the Timm-Haug73 neuropil pattern in the rat brain, except in the olfactory bulb and Islands of Calleja^53,132^, virtually eliminates staining of normal neuronal perikarya^53^ and strongly reduces that of normal glia^133^. As the neoTimm method is designed to de-emphasize the staining of perikarya and glia, aldehyde fixation may be used more freely^53,55,133,134^ than when perikarya and glia are the objects of study. Note, however, that aldehyde fixation combined with sulphide treatment may cause a strong colouring of vessels not seen after sulphide treatment only. The neoTimm version is well suited for studying neuropil undisturbed by the staining of perikarya and glia. A tendency for the original neoTimm version to label the outer third of the molecular layer of the dentate gyrus particularly weakly^53^, while an alternative sulphide administration caused a much stronger staining of this layer^56^, suggests that the original neoTimm version (perfusion with 0.1% sulphide at pH 7.4 for 7 minutes) comes close to under-staining at least this layer of neuropil.

Contrary to the neoTimm method, which was designed to eliminate staining of neuronal perikarya and glia, a methodological study by Brun and Brunk was designed to enhance this staining by increasing the concentration of sulphide ions^127^. Tissue blocks were treated with H_2_S gas and subsequent cryostat-sections were immersed in 1% ammonium-sulphide in 70% ethanol, pH 9.3. This gave a strong granular staining of neuronal somata and glial cells, interpreted as in part representing lysosomes, while the neuropil stain appears to be weak. The concentration of sulphide ions depends on the concentration of sulphide and the pH. A later study^135^ illustrates the importance of pH by showing strong lysosome-like staining with the ammonium sulphide method and very weak such staining after 0.1% sodium sulphide at pH 7.2. Chelation with desferrioxamine supported that the staining in question was caused by iron.

Replacing sulphide by selenium, also in group eight of the periodic table, largely replicates the neuropil pattern seen with sulphide-based versions of Timm’s method, as discussed in great detail for the hippocampal region^60^. As with the neoTimm method, the olfactory bulb is unstained (e.g. Fig. 6a in reference^136^ and Fig. 14a in reference^105^), while neuronal perikarya and glia stain even more weakly than with the neoTimm method^133,134^. Absence of staining in perikarya and glia gives selenium-stained material a very clean look^137–139^ and metal selenides appear to be more resistant to aldehydes than metal sulphides, allowing better preservation of structure in light- and electron microscopy^7,56,57^. Retrograde transport of a zinc-selenium compound is used to map perikarya of zincergic neurons^60,140,141^.

### Chemical interpretation of the staining

Timm’s method has no inherent specificity for a single metal^43^. Here, we shall consider the evidence that the silver grains formed with Timm-Haug73 represent the presence of metals, beyond the case of zincergic boutons, and briefly which metals these may be. Tuning of parameters and solubility tests adapted from inorganic analysis^142^ may enhance selectivity^143^, but the final interpretation requires confirmation by independent methods, as recounted here for the zinc in the Timm-stained, zincergic boutons.

The following observations led to the conclusion that Timm-staining in boutons represents zinc. Initially, Maske identified dithizone staining of “certain parts of the hippocampus” as zinc^144^ by spectrophotometry. Fleischhauer and Horstman confirmed and extended Maske’s morphological observations on dithizone staining to other parts of the telencephalon and other mammalian species, but could not determine the cytological localization of the stain^145^. Timm’s method for zinc and other metals^40^ showed intense staining specifically of the mossy fibre areas, and weaker staining of neuropil in some other parts of the telencephalon^41^. High uptake of zinc in the hippocampal mossy fibre areas^146,147^ supported the hypothesis that the dithizone and Timm-stain is caused by zinc. Evidence accumulated that Timm-staining of the mossy fibre areas and neuropil in other parts of the telencephalon^41,42^ resides in boutons^43,46–52,55,131,148^. Timm-staining of neuropil in other parts of the telencephalon was confirmed to mirror the dithizone pattern^149^. The red dithizone stain was again extracted and identified as zinc dithizonate, although now with a trace of copper^150^. Timm-Haug73-stain of neuropil throughout the telencephalon was blocked by dithizone and diethyldithiocarbamate^151,152^. The Timm-Haug73 and Timm-Danscher81 stain of neuropil in the telencephalon was roughly mirrored by fluorescence microscopy with zinc-specific fluorophores, such as TSQ^38,110,132^. Conversely, TSQ-fluorescence was blocked by sulphide^132^. The final evidence was that zinc-transporter ZnT3 associates with the synaptic vesicles of Timm-stainable boutons^153^, ZnT3 knockout animals are devoid of neoTimm-staining^﻿154,155^, lack zinc-specific fluorescence in the neuropil^38^ and do not accumulate more total zinc in the mossy fibre areas than in adjacent areas^155^. Although the absence of neoTimm staining in ZnT3 knockout animals points to zinc as the *exclusive* basis for Timm-stain in boutons, synaptic roles for copper are claimed^18,156–158^, including even depolarization-induced release from boutons^159,160^.

Whether Timm-staining of neuronal perikarya and glial cells represents metals is discussed below. Whereas pre-treatment with the chelating agents dithizone^151^ and DEDTC^152^ blocked the staining of neuropil (except in the olfactory bulb and Islands of Calleja), it did not block the staining of perikarya and glia. The interpretation was that the Timm-Haug73 staining of perikarya and glia therefore could be artefacts rather than represent metals^53,56,105,161^. However, other chelating agents (1–10-phenanthroline and 2:2-dipyridyl) had already been shown to block the Timm-Haug73 staining of neuronal perikarya and glia in the telencephalon^162,163^ (see also Fig. 16 in reference^53^), indicating that the Timm-Haug73 staining of neuronal perikarya and glial cells do reveal one or more “free” transition metal species. Iron, zinc and copper are the most abundant endogenous transition metals in the normal brain and relevant candidates for this staining. Mechanisms have been identified to handle iron^164^ and copper^165^ in astrocytes, and zinc in glial cells^166^, and astrocytes have a pivotal role in the brain’s transition metal metabolism^164^. This suggests that stores of Timm-stainable metals may exist in glial cells. For the version of Timm’s method by Brun and Brunk^167^, unspecific binding of sulphide was not supported as a source of artefacts, and a coarse-grained staining in neurons and glia, increasing with age, was concluded to represent loosely bound iron in lysosomes. That a similar staining with the Timm-Haug73 version is reduced by storing the sulphide treated cryostat sections overnight, rather than only for about 15 minutes (pp 51,53, reference^43^), is not inconsistent with the higher solubility product of iron sulphides than of zinc and copper sulphides^168^.

Under pathological conditions, zinc-specific fluorophores do reveal a granular staining in neuronal perikarya^37^. This staining is considered to represent zinc which has in part been translocated from synaptic boutons, and in part liberated from intracellular stores (i.e. metallothionein), and may take part in the pathophysiology of ischemic cell death and traumatic brain damage^5^. Whether stronger sulphide treatment, as in Timm-Haug73, may reveal the postulated zinc-stores in normal perikarya has not, to our knowledge, been investigated, nor has the basis for the sulphide-silver labelling of glial cells. Although the concentration of free copper may be “undetectable” in many experimental situations^169^, Timm’s method is claimed to reveal copper stores in normal neurons and glia^142,143,170^.

### Accuracy of image registration

Registration of all images to the WHS rat brain atlas facilitates comparison of morphologies across animals, even when the plane of sectioning differs. The image registration involved affine transformation of customised atlas overlays to match major anatomical landmarks in the histological images^101^, followed by careful adjustment using non-linear transformation and, finally, validation by two independent researchers^171^. In the case of the coronally divided subject H108, different section angles in the anterior and posterior brain block were confirmed and registered with the image registration software, QuickNII. Non-linear transformation of atlas overlay images, defined uing the VisuAlign software, compensated for brain deformations.

The registered atlas overlays are suitable for gross anatomical guidance, while more detailed anatomical investigations could rely on interpretation of the regional patterns in the Timm-stained section and of cytoarchitecture in the adjacent Nissl-stained sections.

## Usage Notes

Here we present examples of how to use the Timm-Nissl atlas. First, we give a summary of the components of this collection including how to interact with the online viewer tool. Next, we exemplify how researchers can use the Timm-Nissl atlas as a useful three-dimensional guide to assist in recording or sampling from precisely defined locations, or to investigate different brain regions. Finally, we conclude with perspectives on the practical value of the atlas as an open resource for the neuroscientific community.

The Timm-Nissl atlas comprises serial microscopic section images from five adult albino (Wistar) rats cut in three different orthogonal planes (coronal, sagittal and horizontal) and stained to reveal both brain metal distribution and cytoarchitecture. The data are available from EBRAINS (https://search.kg.ebrains.eu/) as series of TIFF images for download for each of the 2–3 stainings from five rat subjects (Table 1). Links are provided to view data in an interactive web-microscopy viewer. Inside the LocaliZoom viewer, users can navigate images by zooming and panning, and utilize the optional atlas overlays showing the name, shape and location of brain regions found in the WHS rat brain atlas v4 (Fig. 1) as guidance for interpreting anatomical location. As an additional aid to navigating the large image series H108, Fig. 2 provides an overview of the Timm-Nissl atlas with easy access to all image series in the web-microscopy viewer via embedded links (section images and/or light blue links) in the interactive PDF version of this figure (see Supplementary Figure 1). Figure 2c presents the anteroposterior location of the serial coronal images (H108) relative to major anatomical regions of the WHS rat brain atlas.

Researchers approaching specific areas of the rat brain, may find the regionally differentiated Timm-staining particularly interesting and use the Timm-Nissl atlas as a supplement to the comprehensive literature on their cytoarchitecture, chemoarchitecture and connections. In the following paragraphs we give a few examples of regions to which the present Timm-Nissl atlas may prove a useful three-dimensional guide, whether for allowing a general overview or assisting in recording or sampling from precisely defined locations. More detailed descriptions and illustrations are available in the original publications^43,46,48^.

The morphological divisions of the **olfactory bulb**^172^, visualised by the Nissl-stain, are supplemented by the Timm pattern (Fig. 4a,b). The olfactory nerve layer^173^ (MOBnl^174^, called fibrillary layer in reference^43^) contain densely stained glia extending into the glomerular layer (MOBgl^174^) among the periglomerular cells, as seen at low magnification (Fig. 4b’, see Nissl-stain for demarcation of individual glomeruli). The glomeruli themselves contain finer grains. The outer plexiform layer (MOBopl^174^) is paler than the glomeruli, here staining is associated with several small cells. Mitral and granule cells are covered or filled with large black granules (Fig. 4a,b’, and Fig. 12 in reference^48^).

Researchers interested in the **hippocampal region**^174^ (HR) may find it useful to compare Timm- and Nissl-staining between brains cut in different planes of orientation. The curved longitudinal (septotemporal) axis of the dentate gyrus (DG) and adjacent hippocampal subfields does not align with any standard plane of sectioning, but different viewpoints can aid in the comprehension of its three-dimensional structure. At dorsal levels, coronal sections are often preferred for displaying the *cornu Ammonis* 1–3 (CA1–3) and DG (subject H108, Fig. 4c–e”). At more ventral levels, horizontal sections (subjects H200 and H441, Fig. 4f–h) enable a simultaneous view of all subareas along the mediolateral axis, i.e. from DG, through subiculum (SUB), presubiculum (PrS), parasubiculum (PaS) and the entorhinal cortex (EC; Fig. 4g). A horizontal view is also a good starting point for studies of EC, including mediolateral subdivisions and gradients (Fig. 4), while sagittal sections may be helpful for studies of the dorsoventral extent of this region (subject H201, Fig. 4i–l).

Interlaminar and interareal borders and gradients, reflecting the distribution of zincergic terminals (Fig. 4c–l), may reinforce and supplement subdivisions made with other methods. The boundary between CA3 and the adjacent DG (Fig. 4h) stands out particularly well in Timm-stained sections. Here, intensely stained mossy fibre boutons (Fig. 4g,mf, white arrow) fill the polymorphic layer of the DG (also known as the hilus) and demarcate it from the moderately stained radiatum and oriens layers of the CA3 region^175^. Supplementing classic cytoarchitectural subdivisions in Nissl-stained sections with Timm-staining has also proven to be helpful in delineating some of the borders between the SUB, PrS, PaS and EC (e.g. Figure 4g). Additionally, researchers interested specifically in zincergic terminal fields may note the gradient of decreasing intensity from septal to temporal levels in the stratum radiatum of CA1 (Fig. 4j, white asterisks; Fig. 4k,l), and from dorsal to ventral levels of EC (Fig. 4i,j), which may be related to other differences in chemoarchitecture, connectivity and functions. Several subdivisions of the parahippocampal areas have been put forward, based on analyses of cytoarchitecture, chemoarchitecture and connectivity. For rat, see Boccara *et al*. 2015^176^, and references therein.

Timm-staining of the **cerebellum** differs from that of the telencephalon by not showing any conspicuous regional subdivision. In Timm-stained sections, the cerebellar cortex appears regionally uniform, with a lightly stained molecular layer (Fig. 4n,ML), and intermediately stained granular layer (Fig. 4n,GL). The Purkinje cell perikarya (PL, Fig. 4n, and black arrows Fig. 4n-o”) present with an orange tint and superimposed black granules in moderately stained sections (Fig. 4o’) and appear more distinct after stronger development (Fig. 4n), see also adjacent Nissl-stained section (Fig. 4o”, black arrow). Some of the black grains may be located in Purkinje cell perikarya and others in Bergman glia^43,170^ although this may not be discernible in Fig. 4n.

Researchers interested in the **brainstem**, may use the atlas to discover regional and cellular patterns of Timm-staining while comparing with neighbouring Nissl-stained sections. While stained neuropil dominates the rat telencephalon, stained glial cells and neuronal perikarya dominate the brainstem, with, to some extent, differentiations between regions and nuclei. To present a few examples, a border between a more profusely stained medial and more weakly stained lateral area, seen at low magnification (Fig. 4f), may coincide with the border between the mesencephalon and thalamus. Particularly strong staining of glia and/or neuronal perikarya characterise some nuclei in the hypothalamic region (e.g. section #261 of subject H108) and nuclei of the brainstem (unspecified in the WHS rat brain atlas; see *tegmentum mesencephali* in Haug 1973^43^, pp 35–43). In Fig. 2, one may identify a range of section numbers containing the brainstem (~270–450) and find section number 301 within the interactive web-microscopy viewer by scrolling the filmstrip at the bottom towards the right end (or via a direct link to section number 301 in the interactive PDF version of Fig. 2, see Supplementary Figure 1). Moving through sections onwards to section number 379 as in Fig. 4g and zooming in (Fig. 4m,p-r) reveals large round perikarya (Fig. 4q, black arrows) of the mesencephalic trigeminal nucleus, filled with cytoplasmic granules. Just below (Fig. 4o,r) we have an example of a clearly distinct glial cell, a structure more easily spotted in lightly stained sections (Timm-light; Table 1, H108c and H201b). Glial cells are observed with perikarya and processes of variable size and length, but as black structures.

Other characteristic structures in the Timm-Nissl atlas are the septal nuclei^177,178^ (found within the septal region in the WHS rat brain atlas), basal forebrain region^179,180^, ventral striatum^180,181^ (constituted by the *nucleus accumbens*, ventral pallidum, ventral striatal region in the WHS rat brain atlas), amygdala^138,177,182–184^ (found within the amygdaloid area in WHS rat brain atlas), and the olfactory tubercle. The latter, classified as part of the ventral striatopallidum^180^ (found within the basal forebrain region in the WHS rat brain atlas) has a complex cellular structure^185^, connectivity and chemoarchitecture^186^ and is regarded as a crossroad between olfaction and motivated behaviour^187,188^. Here, neuropil and perikarya are strongly stained with the Timm-Haug73 version, especially the short superficially directed curved stretches of smaller and more tightly packed cells within the cell layer (e.g. section #143 of subject H108). In the so-called Islands of Calleja^189,190^, neuropil and perikarya are intensely labelled.

*In conclusion*, the ability of Timm’s sulphide silver method to stain zincergic terminal fields has made it a useful neuromorphological marker for a class of terminals and thereby suitable for refined subdivision of cortical and other telencephalic areas and studies of development and plasticity in the central nervous system. Comparing Timm-stained features with cytoarchitecture of neighbouring Nissl-stained sections between brains serially sectioned in different planes may help refine definitions of regions and subregions. Researchers planning to investigate a particular region of the rat brain could use the Timm-Nissl atlas to choose optimal planes of sectioning for a given purpose and manoeuver morphological differences between layers, subareas and gradients, e.g. for the purpose of locating points to record or sample from. Researchers using Timm-Haug73 on their own material may use series (of H109, H108 and H201) of different staining intensity to choose staining strengths for their targeted regions and levels of cytological details. We hope the atlas will assist further studies of zincergic fibre systems and inspire research into the nature and functions of “free” transition metals in general, including in neuronal perikarya and glial cells, perhaps focusing on specific areas or nuclei that may be located by means of the atlas. In the EBRAINS Knowledge Graph, the Timm-Nissl atlas images are connected to other relevant datasets and tools via the assigned metadata. Expanding the present collection with more subjects will increase the scientific value and help make this a useful benchmark for navigating and interpreting the normal rat brain.

## Data Availability

The associated files are made available in non-proprietary formats via EBRAINS^102,103^ (https://ebrains.eu/). Section images are shared in standard TIFF format, compatible for display and analyses through a range of tools. The pre-processing and image registration software, Nutil^98^ (RRID: SCR_017183), QuickNII^101^ (RRID: SCR_016854) and VisuAlign (RRID: SCR_017978), as well as the Waxholm Space atlas of the Sprague Dawley rat brain^96,97,99,100^ (WHS rat brain atlas, RRID: SCR_017124), are available for download via NITRC, NeuroImaging Tools and Resources Collaboratory (https://nitrc.org/). Spatial relationships between images and the WHS rat brain atlas, available in JSON files, can be inspected and manipulated with the QuickNII tool presuming that PNG versions of the images is present in the same folder as the related JSON file.

## Supplementary Figure 1

Supplementary materials
