# Supplementary material for: A Timm-Nissl multiplane microscopic atlas of rat brain zincergic terminal fields and metal-containing glia
Source: Sci Data. 2023 Mar 21;10:150. doi: 10.1038/s41597-023-02012-6 (PMC10030855; doi:10.1038/s41597-023-02012-6)
Supplement: Supplementary file 1 — Supplementary materials [file 41597_2023_2012_MOESM1_ESM.pdf]

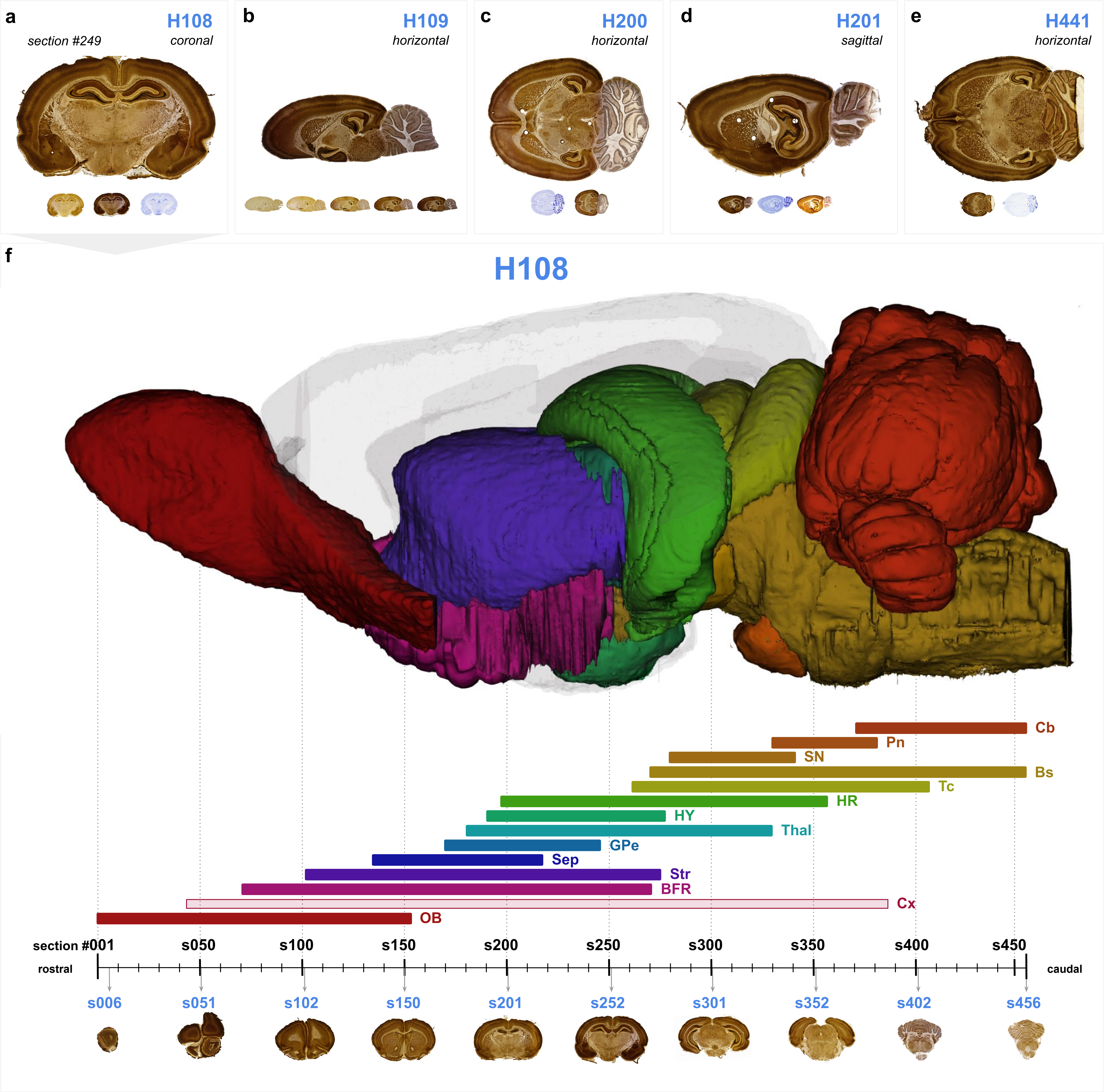

Supplementary Figure 1. Navigating the rat brain Timm-Nissl atlas.

The rat brain Timm-Nissl atlas comprises section images from five rat brains presented in a web-microscopy viewer (via links available from the EBRAINS data card, or via embedded links in this interactive PDF version of the Figure). The content of the (a) coronal (**H108**), horizontal (b) **H109**, (c) **H200**, (e) **H441**) and (d) sagittal (**H201**) series are exemplified with an image of the more intense version of Timm-staining (Timm-dark) as seen in the web viewer. (f) Overview of the rostral to caudal distribution of the coronal image series (H108), relative to surface rendered brain regions in the Waxholm Space atlas of the Sprague Dawley rat brain (WHS rat brain atlas, with custom made colour coding). Colour coded bars indicate the rostrocaudal range of section numbers distributed along the horizontal axis, that include the different brain regions visualised above. Gray numbers indicate the section number and rostrocaudal position of the thumbnail section images shown below. The figure can be used as an initial guide to identify the range of coronal sections that contain a region of interest, e.g. the hippocampal region (HR), which is visible in section numbers 200–360. WHS rat brain atlas v4: BFR, basal forebrain; Bs, brain stem; Cb, cerebellum; Cx, cerebral cortex; GPe, globus pallidus, external segment; HR, hippocampal region; HY, hypothalamus; OB, olfactory bulb; Pn, pontine nuclei; Sep, septal region; SN, substantia nigra; Str, striatum; Tc, tectum; Thal, thalamus.
